# Hybridization produces novelty when the mapping of form to function is many to one

**DOI:** 10.1186/1471-2148-8-122

**Published:** 2008-04-28

**Authors:** Nicholas F Parnell, C Darrin Hulsey, J Todd Streelman

**Affiliations:** 1 School of Biology Institute for Bioengineering and Bioscience Georgia Institute of Technology 310 Ferst Drive Atlanta, GA 30332-0230, USA; 2 Department of Ecology and Evolutionary Biology University of Tennessee Knoxville, TN, USA

## Abstract

**Background:**

Evolutionary biologists want to explain the origin of novel features and functions. Two recent but separate lines of research address this question. The first describes one possible outcome of hybridization, called transgressive segregation, where hybrid offspring exhibit trait distributions outside of the parental range. The second considers the explicit mapping of form to function and illustrates manifold paths to similar function (called many to one mapping, MTOM) when the relationship between the two is complex. Under this scenario, functional novelty may be a product of the number of ways to elicit a functional outcome (i.e., the degree of MTOM). We fuse these research themes by considering the influence of MTOM on the production of transgressive jaw biomechanics in simulated hybrids between Lake Malawi cichlid species.

**Results:**

We characterized the component links and functional output (kinematic transmission, KT) of the 4-bar mechanism in the oral jaws of Lake Malawi cichlids. We demonstrated that the input and output links, the length of the lower jaw and the length of the maxilla respectively, have consistent but opposing relationships with KT. Based on these data, we predicted scenarios in which species with different morphologies but similar KT (MTOM species) would produce transgressive function in hybrids. We used a simple but realistic genetic model to show that transgressive function is a likely outcome of hybridization among Malawi species exhibiting MTOM. Notably, F_2 _hybrids are transgressive for function (KT), but not the component links that contribute to function. In our model, transgression is a consequence of recombination and assortment among alleles specifying the lengths of the lower jaw and maxilla.

**Conclusion:**

We have described a general and likely pervasive mechanism that generates functional novelty. Simulations of hybrid offspring among Lake Malawi cichlids exhibiting MTOM produce transgressive function in the majority of cases, and at appreciable frequency. Functional transgression (i) is a product of recombination and assortment between alleles controlling the lengths of the lower jaw and the maxilla, (ii) occurs in the absence of transgressive morphology, and (iii) can be predicted from the morphology of parents. Our genetic model can be tested by breeding Malawi cichlid hybrids in the laboratory and examining the resulting range of forms and functions.

## Background

Biologists are captivated by the evolution of new forms and functions [1]. Over large evolutionary scales, new traits like oxygen metabolism [2], flowers [3], limbs [4] and the vertebrate neural crest [5] have facilitated the ecological and numerical dominance of the lineages that possess them. On more recent timescales, organisms adapt to their environments by continued innovation and modification of these features. A growing literature seeks to explain the genetic and developmental mechanisms of both macro- and micro-evolutionary novelty [6].

Two recent but largely separate lines of research address the question. The first amounts to a renaissance in our understanding of the evolutionary role of hybridization [7,8]. Hybrid organisms are generally unfit when compared to parent populations [9]. However, hybrids may have greater fitness than their parents in extreme environments [10], when environments fluctuate [11], or after environmental and/or human disturbance [12]. Hybridization contributes to 'creative' evolution in two major ways: by (i) providing hybridizing species with genetic variation for adaptive traits, or (ii) producing new species. Hybrid origin (or hybrid swarm) theories of evolutionary radiation combine these two ideas [13,14]. As an example of (i), Grant *et al*. [15] used long-term study of Darwin's finches to show that directional introgression of alleles from *Geospiza fortis *to *G. scandens*, coupled with natural selection, could explain trends in beak shape and body size. The work of Rieseberg and colleagues on *Helianthus *sunflowers provides one of the best examples of (ii). Hybrid speciation in *Helianthus *is a product of chromosomal rearrangement [16] and ecological selection acting on pleiotropic genes in remote habitats [10]. *Helianthus *hybrid species specialize in extreme environments, where they out-compete their parents [17]. This phenomenon of transgressive segregation (TS), in which hybrids outperform parental forms, is more common than once believed. Rieseberg and others [18] summarized data from 1229 traits in 171 experiments and found that 91% of studies reported at least one transgressive trait and that 44% of traits were transgressive overall.

Importantly, Rieseberg *et al*. [18] also considered the genetic architecture of TS and proposed that complementary action of genes with additive effects was the major cause. Complementary gene action works as follows (see also Table 1 of [18]): species A (large) segregates 6 quantitative trait loci (QTL) for size and 4 of these contribute phenotypic effects of 'large,' while 2 contribute phenotypic effects of 'small;' species B (small) also segregates 6 QTL for size, 4 of which contribute phenotypic effects of 'small,' while 2 contribute phenotypic effects of 'large.' Species A and B produce hybrids that intercross to produce F_2_; after recombination and independent assortment, some F_2 _are larger than parent A (e.g., F_2 _with 6 'large' QTL) and some are smaller than parent B (e.g., those with 6 'small' QTL). Thus, complementary gene action may be a general genetic mechanism of TS.

An upshot of this reasoning was that traits experiencing a history of strong directional selection were less likely to exhibit TS [19]. This was codified in Orr's QTL sign test [20], which compares the distribution of direction of QTL effects to a random expectation, as a quantitative genetic metric of directional selection. Albertson and Kocher [21] extended this logic to argue that directional selection and genetic architecture limit the evolutionary role of TS. Using Malawi cichlid hybrids, they showed that lower jaw shape, a trait under strong directional selection [22], did not exhibit TS while the shape of the neurocranium, a trait not under directional selection, did. They concluded that natural selection might constrain the degree of diversification that can be achieved via hybridization. This might hold, in particular, for aspects of the craniofacial skeleton involved in feeding, including the shape of skeletal components, the dentition, and simple levers involved in jaw function [22-24].

**Figure 1:**
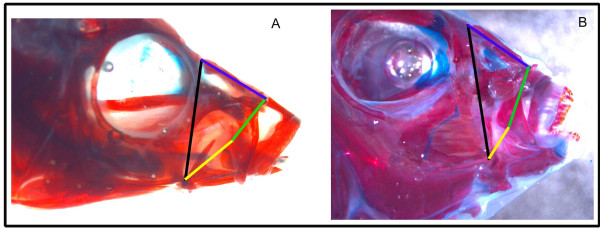
**The components of the 4-bar linkage system are illustrated on cleared and stained cichlid heads of (A) *Copadichromis eucinostomus *(representative high KT) and (B) *Chilotilapia rhoadesii *(representative low KT).** Yellow is the lower jaw (input) link; black is the fixed link, blue is the nasal link and green is the maxillary (output) link. Note differences in relative lengths of input and output links for these high and low KT exemplars.

A second line of research targeting the evolution of novelty departs from a concentration on trait morphology *per se *and instead focuses on function, or more specifically the relationship between form and function. Investigators have contrasted the relationship between shape and biomechanics for simple versus more complex lever systems in fish jaws to illustrate a general principle of organismal design [25]. For instance, the 4-bar linkage system (Figure 1) models the rotation of the maxilla given a known input of lower jaw depression. The output of the model can be computed by geometry when component lengths and angles are measured. As with simpler, two component lever systems, one can estimate kinematic transmission (KT), in this case the amount of motion transmitted to the maxilla, through the linkage system, from lower jaw depression [26]. Jaw linkages with higher maxillary KT transmit more motion through the system and are predicted to evolve in fishes that feed on elusive prey [27]. In contrast to simple levers, morphological variation is only a modest predictor of KT for the 4-bar system [25]. Many different morphological designs can yield similar values of maxillary KT [26]; this is called many to one mapping (MTOM) of form to function. In essence, MTOM is an example of functional redundancy. Diverse lineages that exhibit MTOM may be predisposed to evolutionary novelty, because manifold solutions exist among species for new ecological or functional demands [25]. MTOM of form to function may be a general property of all but the simplest biomechanical systems and may influence system diversity, robustness, modularity and evolvability in complex ways [28].

We reasoned that MTOM of form to function might contribute to *functional transgression *in hybrid offspring. Just as complementary gene action describes hybrid trait transgression as the piling up of QTL of the same direction (e.g., 'large') from different parental genomes, we envisioned scenarios in which MTOM parental species might segregate 4-bar links to hybrids in combinations producing biomechanical output (KT) well beyond the parental range. To evaluate this possibility, we characterized the 4-bar mechanism in Lake Malawi cichlids and simulated hybrids using a simple but realistic genetic model. Lake Malawi cichlids are apposite study organisms for this question. They exhibit tremendous morphological diversity on a background of genomic mosaicism [[29,30], Loh *et al*. 2008 in review]. Species hybridize in the wild [31-33] and speculation persists that hybridization has contributed to the adaptive radiation of this species flock [13]. Our simulations demonstrate that transgressive function is a likely outcome of hybridization among Malawi species exhibiting MTOM. Functional transgression is a product of recombination and assortment between alleles controlling the lengths of the lower jaw and the maxilla, occurs in the absence of transgressive morphology, and can be predicted from the morphology of parents.

## Results and Discussion

### Diversity and correlation in the Malawi 4-bar linkage

**Figure 2:**
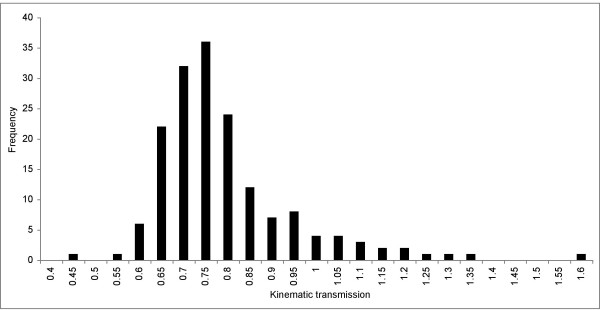
The distribution of kinematic transmission (KT) for 169 individuals from 86 Lake Malawi species shows that the majority of individuals have KT values between 0.65 – 0.80.

We calculated maxillary KT from 86 Lake Malawi cichlid species (169 individual fishes) sampled in July 2005 or borrowed from the American Museum of Natural History. Malawi species exhibit a wide spread of KT values (range for individuals, 0.41 – 1.57; Figure 2; [see Additional file 1 for species averages]) comparable to that observed in other fish lineages [34,35]. The bulk of the specimens had KT values between 0.61 – 0.80 (avg. = 0.77; sd. = 0.16), corresponding to putatively intermediate jaw speeds and force potentials. Planktivores, on average, exhibited higher maxillary KT than other trophic groups [see Additional file 2]. The input (lower jaw, r = 0.473; p < 0.0005) and output (maxilla, r = -0.379; p < 0.0005) links were significantly and antagonistically correlated with KT across the data set (Table 1).

**Table 1:** Correlations (r^2^) between links and KT among Lake Malawi cichlids are similar for uncorrected (below the diagonal) and phylogenetically independent contrasts (above the diagonal).

	LJ	Max	Nasal	Fixed	KT
**LJ**		0.44*	0.00	0.47*	0.06
**Max**	0.33*		0.12	0.53*	0.30*
**Nasal**	0.06	0.08		0.12	0.11
**Fixed**	0.18*	0.54*	0.06		0.07
**KT**	0.23*	0.15*	0.00	0.11	

Underlined values are negative correlations; * indicates statistical significance at p < 0.0005.

We next asked whether correlations among links and KT were robust to the pattern of Malawi cichlid evolutionary history. It has been known for some time that trait correlations should be corrected for phylogeny because related individuals do not represent independent statistical samples [36]. This is a particularly important and difficult problem in the Malawi cichlid assemblage where species are young (e.g., 1000 years, [29]) and phylogenetic resolution is limited [30,37]. Table 1 (above the diagonal) shows evolutionarily independent correlations calculated using the phylogenetic topology shown in Additional file 3, employing an approach described in Hulsey *et al*. [30]. We use a single mitochondrial genealogy because the locus offers more resolution than nuclear genes in this rapidly evolving lineage [30,37]. The corrected correlations are similar to the uncorrected in all but one case. The lower jaw link, positively associated with KT using uncorrected data, is not associated with KT across the Malawi phylogeny. Further inspection of the data suggests that this is due to lack of power to detect a phylogenetic association (i.e., lack of divergent independent contrasts) as most rock-dwelling mbuna have relatively short lower jaws compared to most non-mbuna taxa.

**Figure 3:**
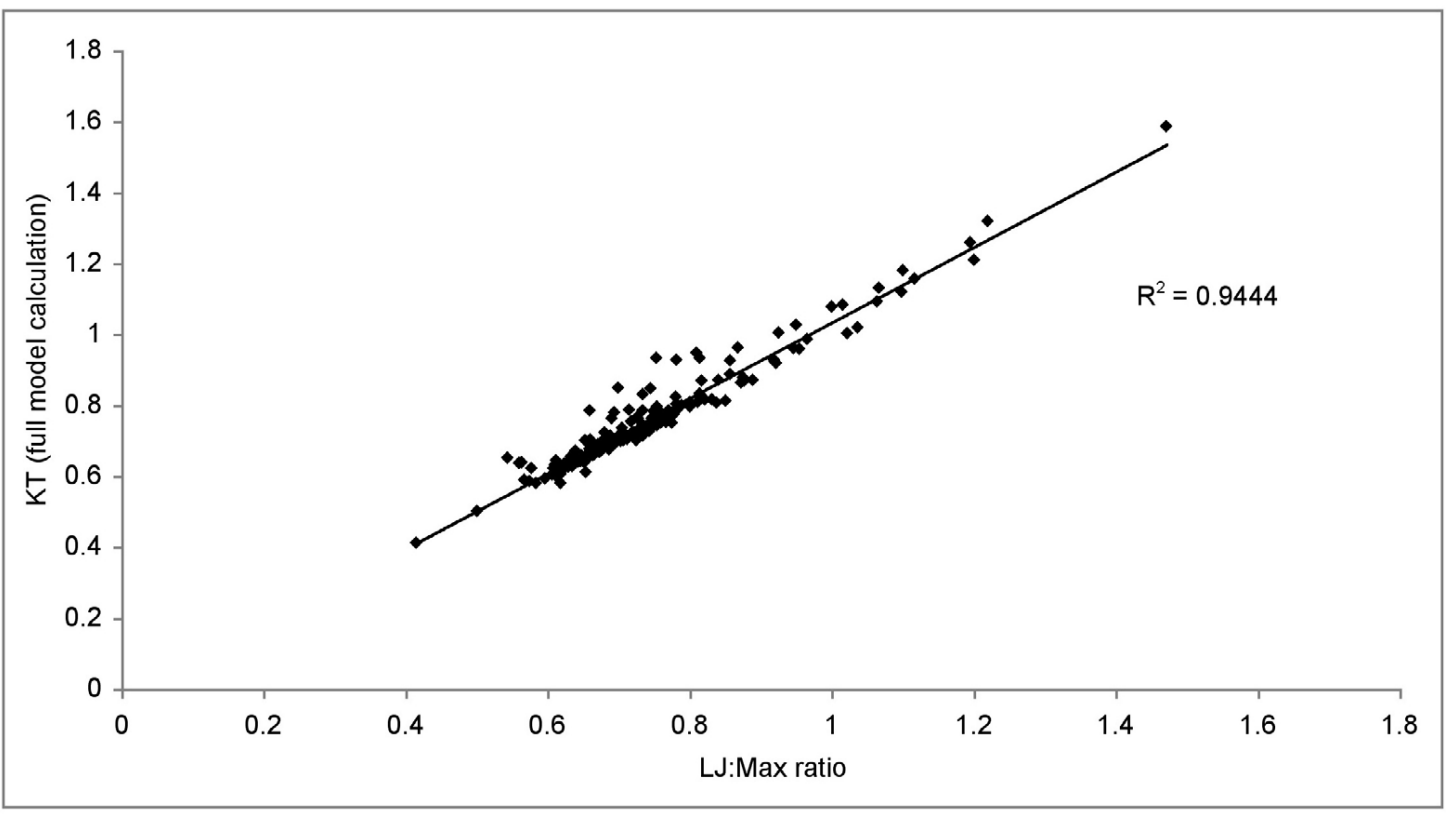
A simple ratio of input (lower jaw) to output (maxilla) links is strongly positively correlated with the fully parameterized calculation of KT.

Given this caveat, our empirical data support results from simulation in other studies [25,26]: longer lower jaws on average yield higher values of KT while longer maxillae produce the opposite (see also Figure 1). Notably, lower jaw and maxillary links were positively correlated with one another in our data set (corrected r^2 ^= 0.44; p < 0.0005; Table 1). Moreover, a simple ratio of LJ/Max lengths was a near perfect predictor of the fully parameterized calculation of KT (Figure 3; r^2 ^= 0.94; p < 0.0005). Our data point to an appreciable linear component to variation in this 4-bar linkage system noted for its non-linear evolutionary dynamics [25,26].

Comparison of species' trait values (corrected for body size) identified numerous cases of convergence in KT via divergence in link length, or MTOM of form to function [25]. The correlations noted above help to explain this observation. For example, both *Cynotilapia afra *and *Pseudotropheus elongatus *have KT values of approximately 0.7, but they exhibit distinct morphologies. *Cynotilapia afra *has relatively long input and output links, while *P. elongatus *has relatively short elements of each. We reasoned that hybridization between species like these that are MTOM for function might produce F_2 _hybrids with extreme KT values, if independent assortment recombines the long lower jaw of *C. afra *with the short maxilla of *P. elongatus *(high KT) or the long maxilla of *C. afra *with the short lower jaw of *P. elongatus *(low KT). We tested the generality of this prediction using a simple, but realistic genetic model.

### Simulated hybrids of MTOM species are transgressive in function

Our Mendelian genetic model assumes no evolution and no environmental variance (V_E _= 0). We specified that size-standardized lengths of each of the four linkage components (Figure 1) were controlled by 4 independent loci (2 alleles at each locus). Links themselves were modeled to be genetically independent of one another. The effect of each allele was assumed to be equal and additive. Importantly, the assumptions of (i) number of loci per link, (ii) genetic independence of links and (iii) additivity of allelic affects are supported by empirical quantitative genetic data for the cichlid craniofacial skeleton [22]. For example, even though the lower jaw and maxilla links are positively (phenotypically) correlated in our data set, this is unlikely to be due to genetic linkage; QTL for the shape of these elements map to distinct chromosomal regions. We used this model to (a) determine if MTOM species would produce hybrids transgressive for function, (b) estimate the percentage of F_2 _progeny exhibiting transgression from specific Lake Malawi cichlid intercrosses, and (c) investigate the genetic contribution of each link to the phenomenon of functional transgression.

**Figure 4:**
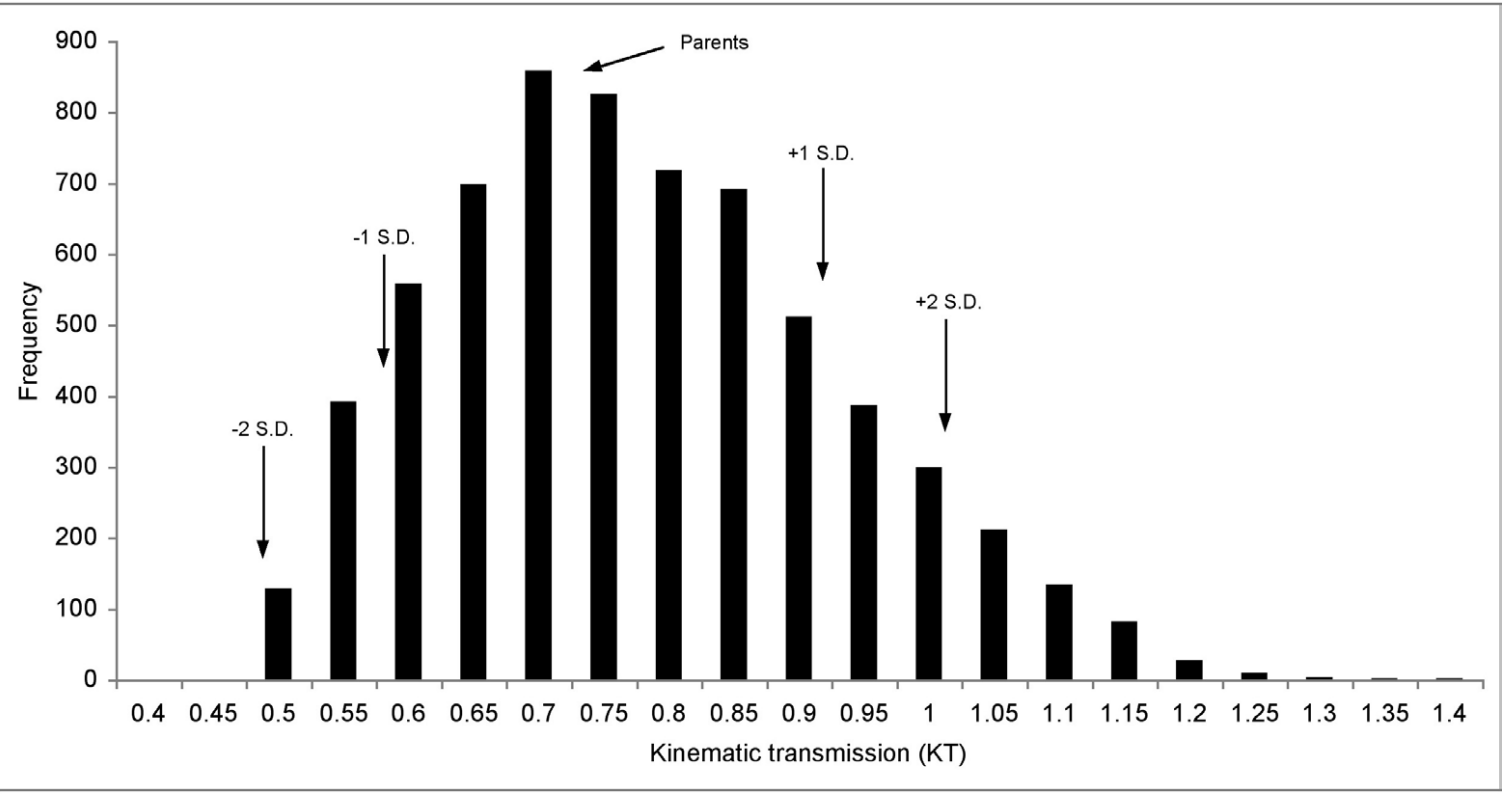
**The distribution of KT values for simulated F_2 _hybrids from the intercross of *Cynotilapia afra *and *Pseudotropheus elongatus *highlights an appreciable frequency (29%) of individuals transgressive for function.** Arrows indicate the KT of the parents and values ± 1 or 2 SD from the mean.

We assumed that each F_0 _parent was homozygous at every locus; since every allele had additive and equal effects, no transgression was produced in the physical lengths of 4-bar links. By contrast, transgression in 4-bar function (KT) was found in 80% of simulated crosses (16/20, Table 2) in which we calculated KT for each and every of 6,561 composite F_2 _genotypes. Note that we have employed a modified and conservative definition of transgression, specific to the distribution of KT produced by the model; an individual is 'transgressive' if its KT value lies ± 1 standard deviation (or more) from the mean. The frequency of transgressive individuals, per cross, ranged from 0.1 – 34%, with up to 6% of the KT values lying two (or more) standard deviations beyond the mean for some (e.g., *C. afra *× *P. elongatus*; Figure 4).

**Table 2:** Simulated crosses of Lake Malawi cichlids produce transgression at appreciable frequencies.

Species	TG F_2_?	% TG
*Cynotilapia afra × Pseudotropheus elongatus*	H/L	29
*Protomelas fenestratus × Protomelas ornatus*	H/L	32
*Chilotilapia rhoadesii × Maravichromis subocularis*	H/L	14.5
*Copadichromis quadrimaculatus × Corematodus taeniatus*	H	1
*Cynotilapia afra × Metriaclima zebra*	H/L	22
*Protomelas ornatus × Protomelas taeniolatus*	H/L	8
*Protomelas spilopterus 'blue' × Protomelas fenestratus*	H/L	21
*Copadichromis virginalis × Chilotilapia rhoadesii*	H	20
*Petrotilapia nigra × Pseudotropheus elongatus*	H/L	29
*Metriaclima zebra × Labeotropheus trewavasae*	H/L	9.2
*Nimbochromis polystigma × Chilotilapia rhoadesii*	H/L	31
*Pseudotropheus elongatus × Labeotropheus fuelleborni*	H	0.1
*Metriaclima zebra × Pseudotropheus elongatus*	H/L	34
*Copadichromis mloto × Otopharynx picta*	H	0.1
*Labidochromis vellicans × Pseudotropheus elongatus*	H/L	19
*Ps. Tropheops 'orange chest' × Ps. Tropheus 'red cheek'*	H	0.1
*Copadichromis quadrimaculatus × Dimidochromis compressiceps*	NO	
*Copadichromis virginalis × Lethrinops altus*	NO	
*Copadichromis virginalis × Tyrannochromis macrostoma*	NO	
*Ps. Tropheops 'orange chest' × Labeotropheus fuelleborni*	NO	

Transgressive (TG) individuals exhibit kinematic transmission (KT) at least one SD either side of the mean from the distribution of hybrid KT values. H/L indicates whether transgression occurs with values higher and lower than the parents.

**Figure 5:**
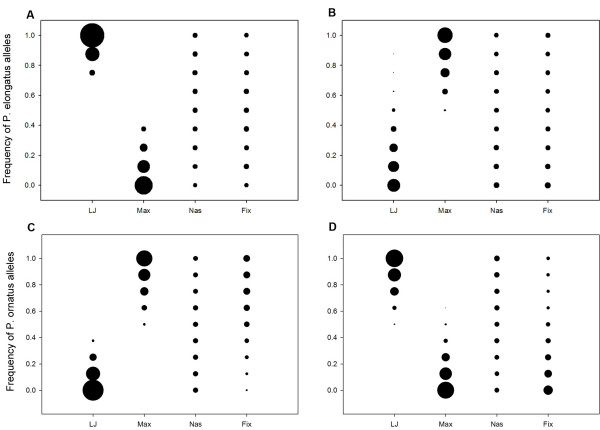
**Bubble plots demonstrate the boundary conditions of transgression for *Cynotilapia afra *× *Pseudotropheus elongatus *(A, B) and *Protomelas fenestratus *× *Protomelas ornatus *(C, D).** For both crosses, transgressive KT lower than (A, C) and higher than (B, D) the parents is dependent on allelic combinations of lower jaw and maxillary links.

We used bubble plots to examine the genetic limits to transgression in each of the simulated crosses and the distribution of those limits among the four link lengths (Figure 5). These plots show the frequency of parental (F_0_) alleles for each link in F_2 _hybrids exhibiting transgressive KT, where hybrid KT is lower (A, C) or higher (B, D) than parental values. For example, in the cross between *Cynotilapia afra *and *Pseudotropheus elongatus*, transgressive KT lower than parental values (Figure 5A) is possible with nearly any combination of alleles for the nasal and fixed links. By constrast, transgression is observed in individuals with certain genotypic combinations of lower jaw (more *P. elongatus *alleles) and maxillary links (more *C. afra *alleles). Bubble plots for the cross between *Protomelas fenestratus *and *P. ornatus *are similar (i.e., negligible influence of nasal and fixed links, importance of lower jaw and maxillary links). These plots can be explained by the starting morphology of the parents (F_0_) and the noted relationship (from simulation and our empirical data above) between the input link (lower jaw), output link (maxilla) and KT. Both *Pseudotropheus elongatus *and *Protomelas fenestratus *have short maxillae and short lower jaws; *Cynotilapia afra *and *Protomelas ornatus *possess long elements of each. Recombination and independent assortment of alleles controlling lower jaw and maxillary links produce functional transgression in KT, according to this model.

**Figure 6:**
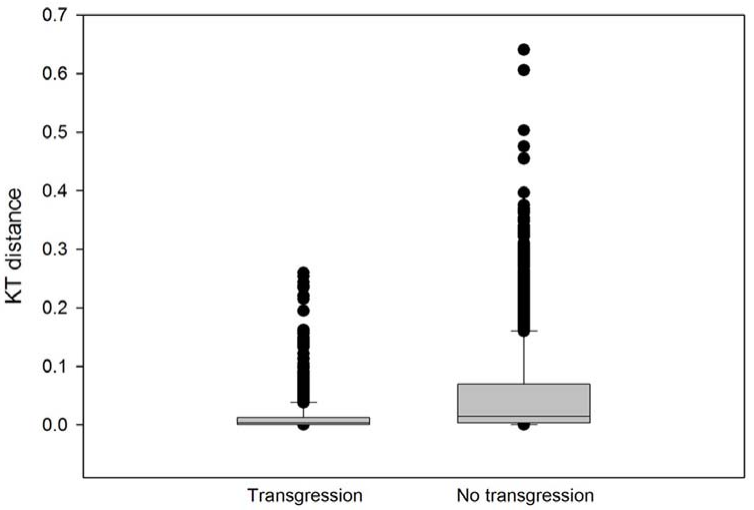
**Box plots demonstrate a relationship between MTOM and transgression.** Crosses that produce transgressive F_2 _are between parental species with less difference in starting KT (a proxy for MTOM) than crosses that do not produce transgressive F_2 _(t-test, p < 0.001). The bar is the median value, the box is the 25^th^-75^th ^percentile, whiskers are the 10^th ^and 90^th ^percentiles and the dots are outliers beyond the 5^th ^and 95^th ^percentiles.

Our choice of the 20 crosses to analyze presents a biased estimate of the proportion of crosses that would yield TS. However, because of the computation involved, a full analysis of all possible crosses in our data set is prohibitive. To overcome this, we realized that the simple ratio of lower jaw length to maxilla length is a strong predictor of KT from the fully parameterized calculation (Figure 3). We also realized, from the detailed analysis of the genetic model above, that transgression is usually produced by recombination between loci specifying the lengths of the lower jaw and maxilla. Combining these ideas, we devised a test to ask whether all pairwise crosses between any two of the 86 species in our data set would produce F_2 _hybrids transgressive for KT. We generated a matrix encompassing all possible pair-wise combinations of size-adjusted lower jaw and maxilla lengths from all species in the data set. Roughly a third of these combinations (29%) produced transgression in KT value. We next used these data to ask whether the occurrence of transgression in F_2 _could be predicted by the degree to which parents (F_0_) are MTOM. As a proxy for MTOM, we used a simple metric of KT distance between the parents of any cross. Formally, if MTOM contributes to hybrid transgression in KT, we expect crosses that produce transgressive hybrids to be between parental species with smaller differences in starting KT. This is in fact what we observed (Figure 6; t-test, p < 0.001).

## Conclusion

We have described a general and likely pervasive mechanism that generates functional novelty. Simulated hybrid offspring among Lake Malawi cichlids exhibiting MTOM are transgressive for function at appreciable frequency. Our modeling approach is noteworthy because it allows us to identify morphological components of the 4-bar mechanical system that contribute to TS. Functional transgression is a product of recombination and assortment between alleles controlling the lengths of the lower jaw and the maxilla and occurs in the absence of transgressive morphology. Transgression occurs when link lengths approaching parental values assort in new combinations in the F_2 _generation. Novel function therefore is realized via new morphological combinations, but not transgressive lengths for any single link. Our model can be tested by breeding Malawi cichlid hybrids in the lab and examining the morphological and mechanical diversity of their 4-bar linkages.

It is important to comment that the model we present shows how MTOM of form to function in the Malawi cichlid 4-bar linkage *could *generate TS in jaw biomechanics. One question raised by the modeling results is why mbuna species have not evolved higher maxillary KT (mbuna range = 0.58 – 0.85). What maintains the positive phenotypic correlation between the lower jaw and maxilla links? One possibility is functional constraint imposed by other aspects of the feeding apparatus. For example, the lower jaw and maxilla both function to position the oral jaws for occlusion and contribute to the gape of the mouth. Mismatches in the length of upper and lower jaw elements may have negative functional ramifications that outweigh increased performance in jaw speed.

### Recombinational evolution, MTOM and the limits to functional diversity

The genomic era has changed the way that evolutionary biologists think about hybridization. Gene flow among recently diverged species (or those evolved through pre-mating barriers) is common rather than the exception [13,15,29]. Hybridization may provide an important source of genetic variation in the form of new trait combinations [10] that sometimes help hybrid organisms maneuver across fitness landscapes [14]. The phenomenon of TS, wherein hybrids outperform the parents, is a frequent observation in laboratory and natural outcrossings [18].

It has been suggested that strong directional selection and simple genetic architecture should limit TS because they point the phenotypic effects of QTL in the same direction; this would essentially constrain diversification by hybridization [21]. Our analysis shows how MTOM promotes TS in function, without transgressive morphology, via recombination and independent assortment. Because multiple morphological solutions exist for any function (i.e., value of KT), MTOM may buffer those constraints, imposed by directional selection, on the extent of mechanical diversity produced by hybridization. As the craniofacial skeleton evolves, functional demands of numerous mechanical systems shape the lengths and linkages of bony elements [28]. MTOM of form to function in each of these systems might facilitate, or perhaps even require, the evolution of diversity.

## Methods

### Specimen information

Cichlid specimens were collected during July 2005 from various sites in southern Lake Malawi. Fishes were labeled with a unique identifier and fixed in 10% buffered formalin solution. Upon returning to the U.S., carcasses were transferred to 70% ethanol for storage. Additional specimens were borrowed from the American Museum of Natural History (AMNH) to supplement the collection. Up to three individuals of each species (depending on the number available) were cleared with trypsin and double-stained using alcian-blue (cartilage) and alizarin-red (bone). This method allows clear visualization of the skeletal components while maintaining skull articulations [27]. Cleared and stained fish were transferred to glycerin for storage.

### Morphometrics and correlation

We measured the anterior jaw 4-bar linkage model as described [26,38,39]. The four physical units of the linkage were quantified on 169 specimens of 86 Lake Malawi cichlid species. Up to three individuals were measured for each species using dial calipers (nearest 0.1 mm) to quantify each link (Figure 1). The four links (lower jaw or input, maxilla or output, nasal or coupler, and fixed or suspensorium) are described in detail, with their respective measurement landmarks, by Hulsey and Wainwright [26]. The linkage measurements were used to calculate the kinematic transmission (KT) of each jaw [27]. Anterior jaw KT is used as an estimate of the speed of motion transmitted through the 4-bar linkage, and in addition, the measurement of KT simultaneously describes the force transmission (FT) through the 4-bar as the reciprocal of KT [26]. The method of KT calculation followed that of Hulsey and Garcia de León [27] and was accomplished using an iterative function in Microsoft Excel^® ^with a starting angle of 15° and an input angle of 30°.

Correlations were calculated between each of the 4-bar links and KT to examine the relationship therein. Because the structural components of the anterior jaw are highly correlated with body size [[26], NFP unpublished] each 4-bar link was corrected for fish standard length (SL) prior to correlation. We used the residuals of linear regression of link length to SL. Felsenstein [36] has shown that correlations may not be statistically independent when compared across taxa with common evolutionary history. Therefore, we calculated phylogenetically independent contrasts following an approach outlined in Hulsey *et al*. [30]. We constructed a phylogeny of 52 Lake Malawi species using sequences from the mitochondrial ND2 gene [see Additional file 1, Additional file 3]. All species were collected from the wild in Lake Malawi from various locations. DNA extractions, PCR parameters, and sequencing followed previously published protocols [30].

The ND2 gene was transformed into its three codon partitions. ModelTest 3.06 [40] was used to identify the most likely model of molecular evolution for each codon partition. Standard Bayesian analyses were executed to find the maximum log-likelihood topology using MrBayes 3.0 [41]. Independent contrast analyses were performed using CAIC [42] in order to assess correlations among the SL-corrected residuals of the four link lengths and among those values and KT. We employed the highest log-likelihood tree from the above Bayesian search to correct for phylogeny [Additional file 3].

### Genetic model of hybrid crosses

Several approaches can be taken to model the generation of novelty in jaw structures and for this project we chose a simple genetic model. Previous studies have examined how 4-bar function changes with different structural configurations, as well as how the 4-bar can evolve based on selection for different KT [25,26,43,44]. Also, Albertson and Kocher [21] have examined the phenotypic distribution of one interspecific Lake Malawi cross in terms of cranial and jaw morphology. For this paper we wanted a model that would not only describe the distribution of genotypes and phenotypes for specific intercrosses, but also allow the incorporation of empirical data from the Lake Malawi system.

We constructed a simple but realistic individual-based Mendelian genetic model, which incorporated assumptions of no mutation, no selection, and no error. We assumed that the size-standardized (below) length of each of the four anterior jaw links was controlled by 4 independent loci with 2 alleles at each locus, for a total of 8 alleles per link. Links themselves were modeled to be genetically independent of one another. The effect of each allele was assumed to be equal and additive, and therefore each of the four lengths (lower jaw, maxilla, nasal, fixed) for each parental (or F_0_) specimen was divided by 8. Note that the assumptions of (i) number of loci per link, (ii) independence of links and (iii) additivity of allelic affects are supported by empirical quantitative genetic data for the cichlid craniofacial skeleton [22]. We controlled for differences in body size among species in the model by dividing each parental link length by SL. In this way, the heritable unit of link length can be thought of as the proportion of SL made up by each link. We also ran models using residuals from linear regression of link length on SL as input variables, and this did not change the outcome.

Using this rubric, each allele inherited from a parent would impart 1/8^th ^of the total length of that link to the offspring. Parents were assumed to be homozygous at all loci. The initial cross among F_0 _produced F_1 _progeny with identical 4-bar lengths (and KT values) equal to the average of the two parents. A second cross within the F_1 _progeny produced all potential parental and recombinant genotypes in the F_2 _generation (6,561 possible). Hybrid link lengths were calculated by the summation of all allelic contributions totaled over the four loci per link. Thus, each composite F_2 _genotype was assigned a 4-link phenotype, and KT was calculated for all. Some recombinant link lengths did not support a functional jaw linkage in some crosses. This result was taken to represent potentially unfit hybrid individuals within a cross and these individuals were left out of further analyses; this effect has been noted in prior simulation [26]. Frequency distributions of F_2 _progeny were created (MS Excel^® ^and SigmaPlot^® ^8.0) to compare hybrid KT values to parental KT. Bubble plots were generated (SigmaPlot^® ^8.0) to examine the contribution of parental alleles to link length in F_2 _exhibiting transgressive KT. A total of 20 interspecific crosses were simulated with this model and F_2 _KT values were examined for evidence of transgression. Species were chosen for these 20 iterations (i) if there is a report of hybridization between them in nature, (ii) if laboratory hybrids have been made, or (iii) if we were particularly interested in the cross from inspection of parental morphometrics.

These 20 simulated crosses present a biased look at the possibility of transgressive function in Lake Malawi cichlid hybrids. The calculations are iterative and time consuming. We wanted an unbiased means to ask if a hybrid cross between any two species would produce F_2 _with transgressive KT. To accomplish this, we made use of the tight relationship between the fully parameterized model of KT and the simpler ratio of input to output links (Figure 3) and the relationship between input link, output link and KT observed in our data (see Results) and from previous simulation [25,26]. We generated a 86 × 86 species matrix containing all possible combinations of size-adjusted lower jaw and maxilla lengths from all species in the data set (7,310 combinations). We essentially asked if recombining the maxilla from one species with the lower jaw from the other (and vice versa) would produce hybrid linkages transgressive in KT. We carried out this operation for all pairs of species in the data set.

## Authors' contributions

All authors conceived of and designed this project. CDH and JTS collected fish and all authors were involved in fish measurements. NFP performed jaw data analyses and genetic modeling of hybrid crosses. CDH collected and analyzed all sequence data and phylogenetically independent contrasts. All authors read and approved the final version.

## Supplementary Material

Additional file 1 Average KT, trophic group and GenBank accession numbers for all species used in this study. Trophic groups are defined as in Hulsey *et al*. [30]. Click here for file

Additional file 2 Planktivores have higher KT, on average, than other trophic groups. Trophic groups are defined as in Hulsey *et al*. [30]; [see also Additional file 1]. The bar is the median value, the box is the 25^th^-75^th ^percentile, whiskers are the 10^th ^and 90^th ^percentiles and the dots are outliers beyond the 5^th ^and 95^th ^percentiles. Click here for file

Additional file 3 Bayesian phylogenetic hypothesis for Lake Malawi cichlids, derived from the mitochondrial ND2 gene, and used to generate phylogenetically independent correlations among link lengths and links with KT. Posterior probability values are given for selected nodes. Click here for file
