# Supplementary material for: Hybridization produces novelty when the mapping of form to function is many to one
Source: BMC Evol Biol. 2008 Apr 28;8:122. doi: 10.1186/1471-2148-8-122 (PMC2386449; doi:10.1186/1471-2148-8-122)
Supplement: Additional file 1 — Average KT, trophic group and GenBank accession numbers for all species used in this study. Trophic groups are defined as in Hulsey et al. [30]. [file 1471-2148-8-122-S1.pdf]

Genus	species	KT	Diet	GenBank no.
Astatotilapia	calliptera	0.80	Omnivore	AF305280
Aulonocara	macrochir	1.00	Planktivore	
Aulonocara	stuartgranti	0.79	Invertivore	EU661720
Buccochromis	heterotaenia	0.66	Piscivore	EU661719
Chilotilapia	euchilus	0.63	Invertivore	EF585280
Chilotilapia	rhoadesii	0.67	Molluscivore	
Copadichromis	eucinostomus	0.99	Planktivore	EF585268
Copadichromis	mbenjii	0.96	Planktivore	EF585255
Copadichromis	mloto	1.08	Planktivore	
Copadichromis	quadrimaculatus	1.21	Planktivore	AF305314
Copadichromis	virginalis	1.38	Planktivore	
Corematodus	taeniatus	0.41	Fin/scales	
Cyathochromis	obliquidens	0.67	Algivore	
Cynotilapia	afra	0.74	Planktivore	EF585264
Cyrtocara	moorii	0.64	Invertivore	AY930089
Dimidiochromis	compressiceps	0.82	Piscivore	EF585267
Dimidiochromis	kiwinge	0.67	Piscivore	AF305322
Docimodus	evelynae	0.83	Cleaner	EF585252
Fossorochromis	rostratus	0.71	Piscivore	EF585281
Genyochromis	mento	0.60	Fin/scales	AF305297
Hemitaeniochromis	urotaenia	0.64	Piscivore	
Labeotropheus	fuelleborni	0.60	Algivore	EF585259
Labeotropheus	trewavasae	0.64	Algivore	EF585283
Labidochromis	gigas	0.74	Algivore	
Labidochromis	vellicans	0.85	Algivore	
Lethrinops	altus	0.78	Invertivore	
Lethrinops	longimanus	0.61	Invertivore	
Maravichromis	anaphyrmus	0.82	Molluscivore	AF305321
Maravichromis	epichorialis	0.60	Molluscivore	
Maravichromis	incola	0.69	Invertivore	
Maravichromis	lateristriga	0.76	Invertivore	
Maravichromis	mola	0.72	Molluscivore	EF585274
Maravichromis	spilostichus	0.70	Piscivore	
Melanochromis	auratus	0.68	Invertivore	AY930069
Metriaclima	aurora	0.67	Omnivore	EF585266
Metriaclima	callainos	0.71	Omnivore	EF585271
Metriaclima	heteropictus	0.71	Omnivore	
Metriaclima	zebra	0.72	Omnivore	DQ093114
Nimbochromis	fuscotaeniatus	0.70	Piscivore	
Nimbochromis	linni	0.75	Piscivore	EF585279
Nimbochromis	polystigma	0.70	Piscivore	EF585262
Nyassachromis	prostoma	1.19	Planktivore	EU661715
Otopharynx	pictus	1.05	Planktivore	EF585254
Otopharynx	heterodon	0.66	Planktivore	EF585278
Otopharynx	lithobates	0.80	Invertivore	

Otopharynx	selenurus	0.74	Invertivore	
Otopharynx	tetrastigma	0.87	Invertivore	
Otopharynx	walteri	0.89	Invertivore	EU661716
Petrotilapia	"fuscous"	0.66	Algivore	
Petrotilapia	nigra	0.71	Algivore	EU661721
Placidochromis	johnstoni	0.99	Invertivore	
Placidochromis	longimanus	0.69	Omnivore	
Placidochromis	milomo	0.78	Invertivore	EF585251
Placidochromis	subocularis	0.75	Invertivore	
Protomelas	annectans	0.65	Invertivore	EU661718
Protomelas	fenestratus	0.67	Invertivore	AF305301
Protomelas	ornatus	0.66	Invertivore	EU661717
Protomelas	similis	0.70	Algivore	EU661714
Protomelas	spilonotus	0.74	Invertivore	
Protomelas	spilopterus "blue"	0.71	Invertivore	EF585253
Protomelas	taeniolatus	0.68	Algivore	AF305302
Protomelas	triaenodon	0.77	Algivore	
Pseudotropheus	crabro	0.72	Cleaner	EF585256
Pseudotropheus	elongatus	0.69	Algivore	EF585272
Pseudotropheus	livingstonii	0.58	Both	EF585273
Pseudotropheus	lucerna	0.78	Algivore	
Rhamphochromis	esox	1.08	Piscivore	AF305252
Rhamphochromis	lucius	0.93	Piscivore	
Rhamphochromis	macropthalmus	0.76	Piscivore	AF305249
Sciaenochromis	ahli	0.79	Piscivore	
Serranochromis	robustus	0.60	Piscivore	
Stigmatochromis	modestus	0.76	Piscivore	
Stigmatochromis	pleurospilus	0.91	Piscivore	
Stigmatochromis	woodi	0.71	Piscivore	AF305299
Taeniochromis	holotaenia	0.65	Piscivore	
Taeniolethrinops	praeorbitalis	0.98	Invertivore	AF305318
Tramitichromis	lituris	0.86	Invertivore	
Trematocranus	microstoma	0.76	Invertivore	
Trematocranus	placodon	0.72	Molluscivore	EF585261
Tropheops	"broad mouth"	0.69	Algivore	EF559101
Tropheops	"orange chest"	0.78	Algivore	EF585275
Tropheops	"red cheek"	0.81	Algivore	EF585265
Tropheops	gracilior	0.63	Algivore	EF585260
Tropheops	microstoma	0.62	Algivore	EF585258
Tyrannochromis	macrostoma	0.61	Piscivore	EF585257
Tyrannochromis	maculiceps	0.79	Piscivore	
