# Supplementary figures and images for: Hybridization produces novelty when the mapping of form to function is many to one
Source: BMC Evol Biol. 2008 Apr 28;8:122. doi: 10.1186/1471-2148-8-122 (PMC2386449; doi:10.1186/1471-2148-8-122)

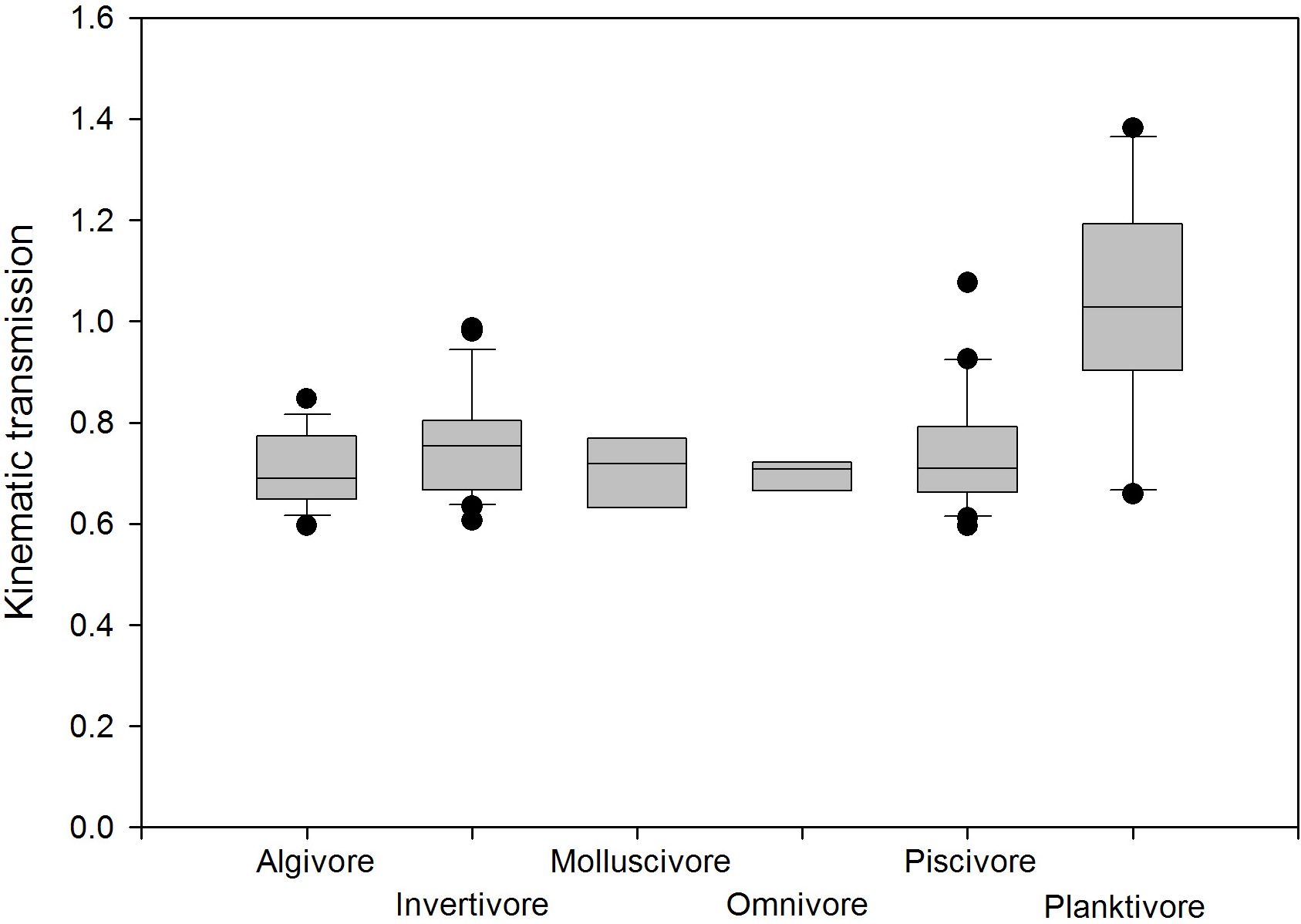

Supplement: Additional file 2 — Planktivores have higher KT, on average, than other trophic groups. Trophic groups are defined as in Hulsey et al. [30]; [see also Additional file 1]. The bar is the median value, the box is the 25th-75th percentile, whiskers are the 10th and 90th percentiles and the dots are outliers beyond the 5th and 95th percentiles. [file 1471-2148-8-122-S2.jpeg]

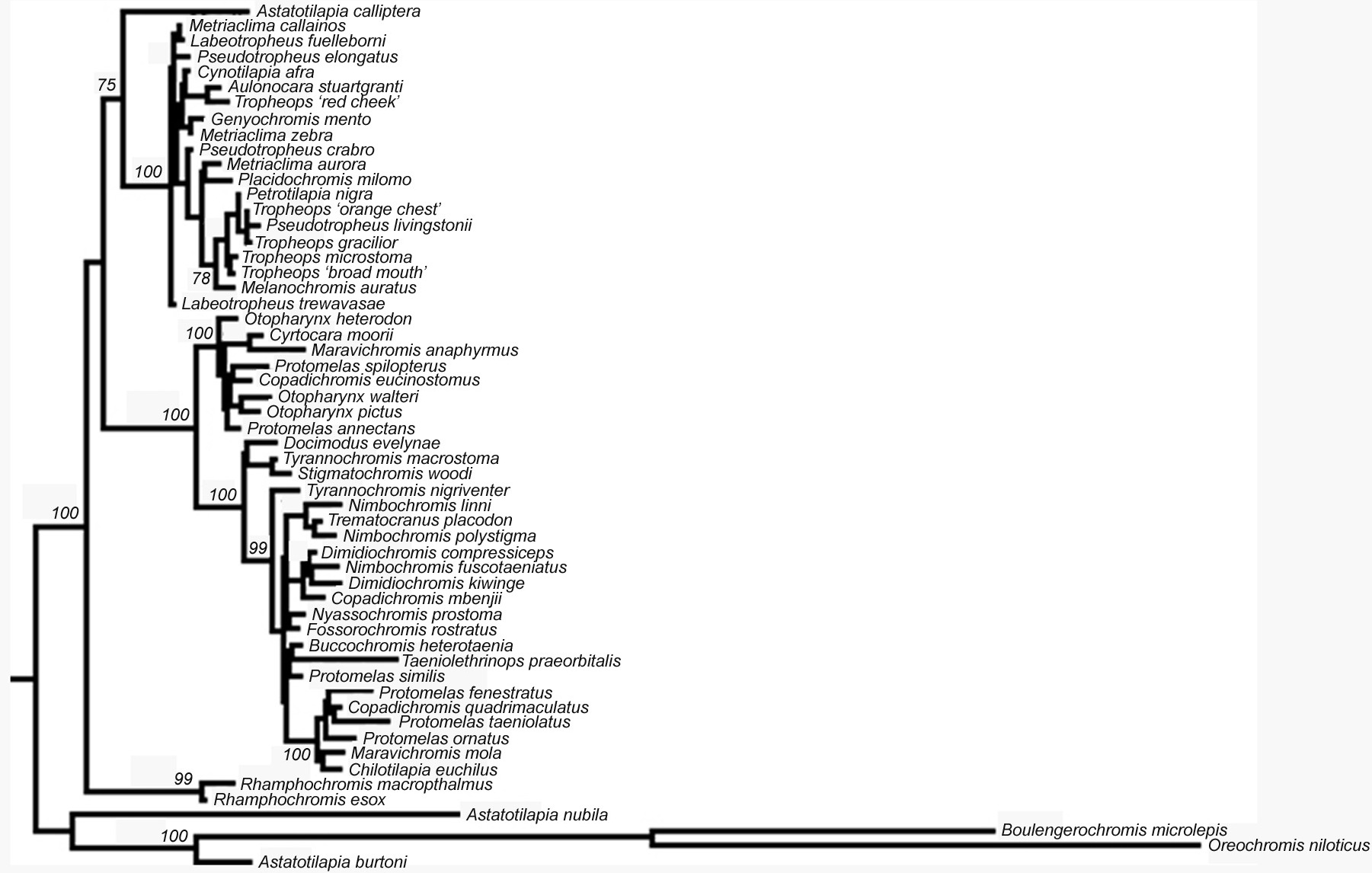

Supplement: Additional file 3 — Bayesian phylogenetic hypothesis for Lake Malawi cichlids, derived from the mitochondrial ND2 gene, and used to generate phylogenetically independent correlations among link lengths and links with KT. Posterior probability values are given for selected nodes. [file 1471-2148-8-122-S3.jpeg]
